# Tumor location determines midkine level and its association with the disease progression in colorectal cancer patients: a pilot study

**DOI:** 10.1007/s00384-012-1476-9

**Published:** 2012-05-06

**Authors:** Malgorzata Krzystek-Korpacka, Dorota Diakowska, Krzysztof Grabowski, Andrzej Gamian

**Affiliations:** 1 Department of Medical Biochemistry, Wroclaw Medical University, ul. Chalubinskiego 10, 50-368 Wroclaw, Poland; 2 Department of Gastrointestinal and General Surgery, Wroclaw Medical University, Wroclaw, Poland; 3 Wroclaw Research Center EIT+, Wroclaw, Poland

**Keywords:** Midkine, Colorectal cancer, Lymph node metastasis, Cytokine, Growth factor

## Abstract

**Purpose:**

The purpose of this study was to evaluate midkine, multipotential cytokine, and growth factor in colorectal cancer (CRC) stratified by tumor location.

**Methods:**

Midkine was assessed immunoenzymatically in paired cancerous and noncancerous tissues from 53 CRCs and referred to CRC stage, tumor location, and size, and circulating cytokine levels.

**Results:**

Midkine was higher in cancerous versus noncancerous tissue in 98 % cases (424.2 vs. 31.1 pg/mg, *p* < 0.0001). Mean fold increase was 30.1; in 72.5 %, the relative increase was over fivefold. Midkine upregulation was more pronounced in colon than in rectum (fold increase: 36.6 vs. 12.7, *p* = 0.005) due to higher midkine level in noncancerous rectal than colonic tissue (45.5 vs. 26.2 pg/mg, *p* = 0.074). Tumor location affected midkine association with CRC stage. Midkine fold change was higher in advanced stages of rectal cancers (16.8 vs. 5.3, respectively in III/IV vs. I/II, *p* = 0.013), while it tended to be lower in colonic ones (25.3 vs. 47.8, *p* = 0.134). In addition, fold change in midkine level was higher in rectal N1 than N0 cancers (17.3 vs. 16.5, *p* = 0.032), while it tended to be lower in colonic cancers (23.6 vs. 50.1, *p* = 0.085). Midkine negatively correlated with tumor size (*r* = 0.40, *p* = 0.017), while it tended to positively correlate with its serum levels (*r* = 0.45, *p* = 0.081).

**Conclusions:**

Midkine is differently expressed in tumors arising from colonic and rectal mucosa, where it may play diverse roles in carcinogenesis. High midkine expression in noncancerous rectal mucosa might contribute to, a characteristic for rectal cancers, higher incidence of local recurrence. Divergent expression of midkine and its association pattern ought to be taken into account while designing midkine-directed therapies for CRC.

## Introduction

Colorectal cancer (CRC) is a malignant tumor arising from the inner wall of large intestine. Worldwide, it is the third cause of cancer deaths in women and fourth in men and the second and the third most frequently diagnosed cancer in women and men, respectively. Unlike in USA and Western Europe, CRC incidence in Eastern European countries is increasing probably as a result of “westernization” of a lifestyle [1].

Individual segments of the colon differ with respect to their embryological origin, innervation, blood supply, lymphatic drainage, histology, physiological function, and content. It seems logical that tumors that arise from proximal and distal colon or, taking into account differences in histology and metabolism, from proximal and distal colon and rectum ought to be treated as distinct entities [2]. Indeed, it has been increasingly recognized that tumor location determines risk and protective factors, mechanisms promoting the disease progression, recurrence pattern, prognosis, and effectiveness of various treatment strategies [2–5]. Unveiling differences in cancer-related molecular patterns with respect to tumor location might aid future targeted therapies for CRC.

Midkine is a multifunctional cytokine predominantly expressed during embryogenesis, while in adult organisms, its expression is restricted to several organs, intestine, among others. However, midkine expression is resumed during inflammation, tissue repair, and carcinogenesis. Midkine displays a number of activities that might be relevant for cancer development, e.g., it has been demonstrated to act as a mitogen, an antiapoptotic, and angiogenic factor, a chemoattractant and haptotactic factor, an immunomodulator, and an inductor of synthesis of several cytokines and growth factors, such as IL-8, TGF-β, MIP-2, and MCP-1 [6, 7]. Data on midkine in CRC are scanty and focused on its involvement in colonic neoplastic transformation [8–12], while data on cytokine levels in CRC are missing. Possible midkine contribution to divergence in molecular patterns involved in promoting tumor progression between CRC with different location has not been evaluated before as well. Hence, the aim of our study was to examine protein level of midkine in tumor versus normal colorectal tissue and relate it to the disease progression, tumor location, and circulating levels of cytokine.

## Materials and methods

### Patients

A group of 53 patients admitted between 2005 and 2008 to the Department of Gastrointestinal and General Surgery of Wroclaw Medical University for curative resection of histopathologically confirmed adenocarcinoma of the colon has been enrolled in current study. Open colectomy (right hemicolectomy *n* = 10; transverse colectomy *n* = 7; left hemicolectomy *n* = 3; sigmoidectomy *n* = 19; rectectomy *n* = 14) was performed in 27 men and 26 women, mean age of 67.8 (range, 36–87). Resected tumors were staged pathologically according to the guidelines of the UICC TNM [13] system. There were 11 cases of T2, 10 cases of T3, and 30 cases of T4, and in two cases, T was not established (Tx); there were 25 cases of N0, 22 cases of N1, three of N2, and three of Nx; no distant metastases were found in 47 cases, hepatic metastases in five cases, and in one, M status was uncertain. When grouped, we had eight patients with stage I, 17 with stage II, 20 with stage III, five with stage IV, and in three, the disease stage could not be assessed. The tumor was located in rectum in 14 cases, in colon in 36 cases (in ascending colon in seven, in transverse colon in seven, in descending colon in three, and in sigmoid colon in 19 cases), and in cecum in three cases. Two patients were excluded from the analysis following pathological examination owing to cancerous tissue present in resection margins.

The study protocol was approved by the Medical Ethics Committee of Wroclaw Medical University, Wroclaw, Poland, and the study was conducted in accordance with the Helsinki Declaration of 1975, as revised in 1983, and an informed consent has been obtained from all patients.

### Analytical methods

A paired tissue samples, one representing tumor and the other noncancerous tissue from the same location (resection margin), have been collected postoperatively for each patient, snap frozen, and stored at −45°C until examination. Frozen tissues (∼0.37 g) were placed in 10 mM Tris–HCl, pH 7.2 buffer 1:2 (*w*/*v*) and homogenized using a Potter homogenizer. Following centrifugation (10 min, 1,850 × *g*, 4°C), supernatants were collected and used for subsequent midkine and protein evaluations. Additionally, serum samples (blood collected by venipuncture prior to surgery, clotted for 30 min, and centrifuged 10 min, 900 × *g*, RT) were available for a subset of 19 patients. Midkine concentrations in tissue homogenates and serum samples were measured with by an enzyme double-antibody indirect immunoassay using Human Midkine ELISA provided by Biovendor (Brno–Modřice, Czech Republic) according to manufacturer’s instructions. An intra-assay coefficient of variation (CV) for this assay is 4.5 % and inter-assay CV is 6.3 %, while the test sensitivity is 33 pg/ml. Protein concentration in tissue homogenates was measured using Bradford method [14] with Bio-Rad Protein Assay (Bio-Rad Laboratories GmbH, Munchen, Germany) with bovine serum albumin as the reference. Midkine concentration was expressed in picograms per milligram of total protein.

### Statistical analysis

Data distribution was tested using the Kolmogorov–Smirnov normality test and homogeneity of variances using Levene’s test. Data on midkine in cancerous tissue were normally distributed, while those in adjacent noncancerous tissue required logarithmic transformation; both are presented as means with 95 % confidence interval. Differences in midkine level between cancerous and normal tissues were analyzed using *t* -test for paired samples. Differences in midkine level or fold change in groups stratified by the disease stage or tumor location were examined using *t* test for independent samples (with Welch correction in case of unequal variances) and one-way ANOVA. Correlation analysis was conducted using Pearson correlation (*r*) test, following removal of outlying observations detected using Tukey test (two outliers detected). All tests were two-sided and *p* ≤ 0.05 were considered statistically significant. Entire statistical analysis was conducted using MedCalc® version 12.1.0.0 (Mariakerke, Belgium).

## Results

### Midkine in normal and tumor tissue

**Fig. 1:**
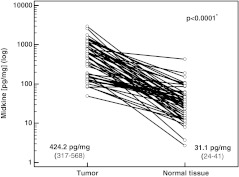
Midkine level in cancerous and normal colon tissue in patients with colon cancer. Midkine level is expressed in picograms of midkine per miligram of total protein (means with 95 % confidence intervals given in *parentheses*). *Asterisk* statistically significant

We compared midkine level in paired cancerous and normal tissue from patients with colon cancer and found mean midkine level to be significantly higher in cancerous tissue (Fig. 1). Except for one case, midkine was higher in tumor than in normal tissue, i.e., in 98 % of the cases. In 37 out of 51 (72.5 %), the relative increase was over fivefold. Mean fold increase in midkine level was 30.1 (18.5–41.7).

### Midkine level and the disease progression

**Fig. 2:**
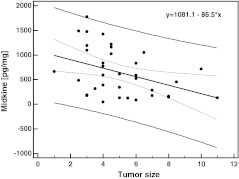
Midkine relation to tumor size (regression line). Data on tumor size available for 36 patients; two outlying observations removed prior to analysis. *R* ^2^ = 0.16, *p* = 0.017. *Thick line* regression curve, *thinner lines* 95 % prediction interval curves, *scattered lines* 95 % confidence interval

Midkine level in cancerous tissue is negatively correlated with tumor size (*r* = −0.40, *p* = 0.017; regression line is presented in Fig. 2) and shows tendency to positively correlate with serum midkine concentrations (*r* = 0.45, *p* = 0.081). There were no significant differences in midkine level between T2, T3, and T4 tumors (*p* = 0.754), in N0 vs. N1 disease (*p* = 0.732), in M0 vs. M1 disease (*p* = 0.485), and among stages I, II, III, and IV (*p* = 0.655).

### Midkine level and tumor location

**Fig. 3:**
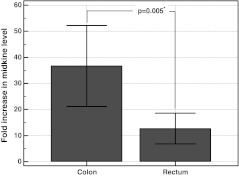
Differences in fold increase in midkine level between cancerous and normal tissues in relation to tumor location. Whiskers indicate 95 % confidence interval. *Asterisk* statistically significant

We observed higher midkine concentration in normal tissue derived from rectum [45.5 pg/mg (26.5–78.3) than from colon (26.2 pg/mg (18.9–36.4), *p* = 0.074]. When cancerous tissues from rectum and colon were compared, no differences in midkine concentration were found (*p* = 0.797). Consequently, fold increase, i.e., ratio of midkine in cancerous to noncancerous tissue, was significantly higher in colon than in rectum (Fig. 3). Within colon, however, no difference between proximal and distal part could be observed, both when normal (*p* = 0.862) or cancerous tissues (*p* = 0.839) were compared. The tendency towards higher fold change in midkine level in proximal than distal colonic tumors was not significant (45.6 times vs. 33, *p* = 0.859).

### Midkine level and the disease progression in relation to tumor location

**Fig. 4:**
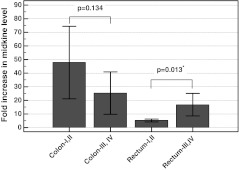
Fold changes in midkine level between cancerous and normal tissue in relation to tumor location and the disease stage. *Whiskers* indicate 95 % confidence interval. *Asterisk* statistically significant

**Fig. 5:**
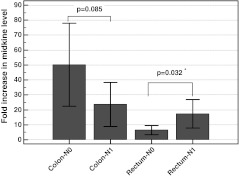
Fold changes in midkine level between cancerous and normal tissue in relation to tumor location and lymph node involvement. *Whiskers* indicate 95 % confidence interval. *Asterisk* statistically significant

We re-examined the data by analyzing the fold changes in midkine level separately for colon and rectal cancers. The fold change in midkine was significantly higher in advanced stages of rectal cancer, while the opposite trend was observed in colonic cancers (Fig. 4). In addition, fold change in midkine level was higher in rectal N1 than N0 cancers, while it tended to be lower in colonic cancers (Fig. 5). Similar tendency was found concerning T stage, where fold changes in midkine tended to be higher in T3/T4 than T2 rectal [15.6 (7–24) vs. 7 (0.9–14), *p* = 0.152] but not colonic tumors [37 (18–56) vs. 41 (5.6–77), *p* = 0.853]. The number of patients with M1 disease was insufficient to allow for the statistical analysis of tumor-location stratified groups.

Difference in midkine association with the disease progression between rectal and colonic cancers was also observed, although less pronounced, when midkine levels in cancerous tissue and not fold changes were compared. Midkine level was significantly higher in advanced stages of rectal [636.5 pg/mg (314.5–1288) in III/IV vs. 190.8 pg/mg (62–588) in I/II, *p* = 0.036] but not colonic cancers [respectively 409.5 pg/mg (227–740) vs. 514.5 pg/ml (308–858), *p* = 0.551]. It tended to be higher in N1 vs. N0 disease in rectal [580 pg/mg (266–1265) vs. 264.2 pg/mg (80–871), *p* = 0.180] but not in colonic cancers [375 pg/mg (212–664) vs. 553 pg/mg (330–928), *p* = 0.301]. Furthermore, midkine level tended to be lower in T3/T4 than T2 colonic tumors [respectively 399 pg/mg (265–601) vs. 833 pg/mg (475–1459), *p* = 0.146] but not rectal tumors [465 pg/mg (193–1117) vs. 336 pg/mg (98–1151), *p* = 0.606].

## Discussion

To best of our knowledge, this is the first paper showing differences in midkine level as well as in its association pattern with the disease progression between cancers of colon and rectum.

Data on midkine in CRC are surprisingly scanty. Aridome et al. [8] were first to detect, semi-quantitatively, higher midkine expression in cancerous than noncancerous tissue from 12 out of 13 CRC patients (>92 %). Subsequently, Miyashiro et al. [9] confirmed midkine messenger RNA expression in cancerous tissues, either as a full-size midkine or its truncated form, devoid of its N-terminal domain. Since the truncated midkine has been found in cancerous colorectal tissue and in metastatic lymph nodes, but not in adjacent noncancerous tissue, the cancer-specific nature of the truncated form has been suggested [10]. Corroborating with these original findings, we found higher midkine concentration in cancerous than noncancerous tissue in 98 % of examined cases. Owing to the polyclonal character of the antibody used for midkine detection, we were likely to detect both full and truncated midkine forms.

Ye et al. [11] and Tokuyama et al. [12] demonstrated midkine expression to be higher in adenomas and adenocarcinomas than in normal mucosa, being significantly elevated already in low-grade [12] or moderate-grade dysplasia [11]. While Tokuyama et al. [12] observed midkine expression to increase in a stepwise manner, Ye et al. [11] found the highest midkine levels in adenomas. Midkine expression has corresponded with cell proliferative zone, that is, the labeling of Ki-67, a proliferative index [12]. Taken together, these results may imply that elevated midkine expression can contribute to early stages of colon carcinogenesis by facilitating tumor cell proliferation. The issue of midkine expression in relation to CRC stage has not been examined in earlier studies. Supporting the thesis on midkine involvement in early colon carcinogenesis and its possible downregulation in advanced cancers, we found a negative correlation between midkine concentration and tumor size but did not observed any other association with the disease advancement. However, further analysis revealed that midkine expression differed with respect to tumor location. Not only we found midkine to be expressed more profusely in noncancerous rectal than colonic tissue, but also tumor location determined midkine association with the disease stage. Midkine was upregulated in advanced cancers in rectum, being significantly associated with overall disease stage and lymph node involvement, but downregulated in advanced colonic cancers. Previously, Song et al. [15] observed serum midkine to be more frequently elevated in lymph node positive than negative colon cancers. The association of circulating midkine with lymph node metastasis has also been demonstrated in esophageal squamous cell carcinoma [16] and in endometrial cancers [17]. Our results, if confirmed in a larger cohort, might suggest divergent role of midkine in colonic and rectal carcinogenesis.

A body of evidence has recently been gathered documenting variability in molecular patterns of colorectal cancers located in various parts of large intestine. Proximal colonic tumors have been observed to overexpress Ki-67 and p53 [4], Bcl-2 (solely in female) [4], keratins, and carbonic anhydrases [18] while in distal colonic tumors COX2 and teratocarcinoma growth factor [18]. Tumors of proximal colon are also more likely to be poorly differentiated [4, 5, 19] and associated with worse overall but not cancer-specific survival [5]. In turn, Derwinger et al. [5] reported differences within distal colonic cancers with cancers of sigmoid colon having more favorable prognosis expressed in terms of overall survival and cancer-specific survival, resulting from better stage and grade, as compared to descending colon. Correspondingly, fold change in midkine in descending colon tumors was higher than in sigmoid colon tumors, but as we examined only three cases of the former, the difference did not reached statistical significance (45 times vs. 31, data not shown). Minoo et al. [19] examined differences in expression patterns of 50 markers associated either with major signaling pathways involved in tumor progression or with immune response, of which several were more frequently overexpressed in rectal than other distal colon cancers (CD44v6, E-cadherin, CD68, CD163, and Foxp3), while others were found to be differently expressed in rectal vs. proximal cancers (TOPK, APAF-1, p21, Foxp3, TIA-1, CDX2, and β-catenin). In turn, Kalady et al. [20] found a higher incidence of microsatellite instability, methylator phenotype, and mutations in the oncogene *BRAF* in colonic than rectal cancers. Microsatellite stable tumors had almost four times higher risk of disease recurrence [20], what may contribute to higher incidence of local disease recurrence and account for generally worse prognosis characteristic for rectal cancers. In this respect, higher baseline midkine expression in rectal mucosa together with a direct midkine association with disease progression might contribute to this feature of rectal cancers. It is worth mentioning that midkine has recently been suggested as the recurrence marker for desmoid tumors, monoclonal neoplasms that may occur as a part of familial adenomatosis polyposis [21].

Midkine overexpression is common among solid tumors, as it may facilitate tumor cell proliferation, survival, and migration, and enables invasion. As such, midkine might become a promising target for future directed therapies. A number of strategies to inhibit midkine expression/signaling are under investigation [22], and successful suppression of hepatocellular carcinoma growth by midkine-antisense oligonucleotide-loaded nanoparticles has already been reported [23]. However, regional divergence in midkine expression and, possibly, the role it might play in the disease progression ought to be taken into account while designing midkine-directed therapies for CRC.

Taking into account regional differences in midkine level, relative changes expressed in terms of fold changes rather than absolute cytokine levels should be evaluated in future studies. However, it would be of interest to compare midkine level in colonic and rectal tissues from noncancer patients to address the issue whether higher midkine level in noncancerous rectal than in colonic mucosa observed in CRC is disease related.
